# Recent Investigations on Pathogenesis, Biomarkers, Epigenetics, and Emerging Therapeutic Strategies to Modulate Multiple Signaling Pathways in Diabetic Retinopathy

**DOI:** 10.1155/joph/5539383

**Published:** 2026-05-13

**Authors:** Zixuan Huang, Jing Wei, Dayong Yang

**Affiliations:** ^1^ Department of Ophthalmology, Affiliated Hospital of Inner Mongolia Medical University, Hohhot, 010050, China, nmgfy.com

**Keywords:** biomarkers, diabetic retinopathy, emerging therapies, epigenetics, lipoproteins, neurovascular interactions, oxidative stress, systemic inflammation

## Abstract

**Background:**

Diabetic retinopathy (DR) is a progressive microvascular complication related to diabetes mellitus and remains a profound cause of vision impairment and blindness globally. In addition to its ocular manifestations, DR is increasingly recognized as a main risk factor for systemic diseases, including cardiovascular and neurodegenerative disorders. Despite advancements in screening and management strategies, challenges persist in evaluating DR progression, identifying reliable biomarkers, and developing targeted therapies. Recent insights into the role of lipoproteins, epigenetics, and systemic inflammatory pathways in DR pathogenesis have opened new avenues for research and therapeutic interventions.

**Objective:**

This review aims to provide a comprehensive update on the pathogenesis of DR, highlighting emerging biomarkers and epigenetic factors involved in disease progression. In addition, the efficacy of novel therapeutic approaches targeting key signaling pathways in both preclinical and clinical settings is explored.

**Methods:**

A comprehensive literature review was conducted using multiple public databases, including PubMed, Scopus, Google Scholar, and the National Library of Medicine. Relevant studies published in the past decade were analyzed to assess advancements in DR pathophysiology, biomarker discovery, epigenetic implications, and emerging therapeutic strategies. Key findings were synthesized to present an integrated perspective on the evolving landscape of DR research and management.

**Results:**

Our analysis reveals that DR pathogenesis involves complex neurovascular interactions, oxidative stress, and hyperglycemia‐induced epigenetic modifications. Lipoprotein metabolism and menopausal hormonal changes were found to influence DR progression, emphasizing the need for personalized risk assessments. The review further highlights the role of circulating blood biomarkers, omega‐3 fatty acids, microRNAs, and inflammatory cytokines as potential indicators of disease severity. In addition, novel therapeutic approaches, including anti‐VEGF agents, neuroprotective strategies, and epigenetic modulators, have shown promising results in preclinical and early clinical studies. Targeting multiple signaling pathways, including VEGF, Wnt/β‐catenin, and inflammatory cascades, holds significant potential for improving DR outcomes.

**Conclusion:**

Advancements in biomarker research and epigenetic regulation provide significant avenues pertinent to DR pathogenesis and progression. The integration of molecular and clinical approaches is essential for developing targeted therapies that address both ocular and systemic implications of DR. Future research should focus on translating these findings into personalized treatment strategies to enhance patient outcomes and prevent vision loss.

**Highlights**
• Comprehensive analysis of DR pathogenesis, including neurovascular interactions and oxidative stress.
• Identification of novel blood biomarkers and epigenetic regulators implicated in DR progression.
• Exploration of menopause‐associated lipoprotein changes and their impact on DR risk.
• Systemic implications of DR in cardiovascular and neurodegenerative diseases.
• Emerging therapeutic modalities targeting VEGF, Wnt/β‐catenin, and inflammatory pathways.
• Future perspectives on personalized treatment strategies integrating molecular and clinical findings.
• Novelty statement: This review provides an advanced perspective on the interplay between systemic factors, epigenetic modifications, and DR pathogenesis. By bridging molecular mechanisms with clinical applications, it offers a unique synthesis of current research trends, biomarker developments, and innovative therapeutic strategies. The integration of neurovascular interactions and systemic metabolic influences in DR management represents a novel paradigm that can reshape future diagnostic and treatment approaches.

## 1. Introduction

Diabetic retinopathy (DR) represents the mostly occurring microvascular complication pertinent to diabetes mellitus (DM) and can be a primary contributor to vision impairment and blindness among adults worldwide [1]. As a progressive neurovascular disorder, DR is hallmarked by a series of pathological changes within the retinal microvasculature, including increased vascular permeability, endothelial dysfunction, pericyte loss, capillary nonperfusion, microaneurysm formation, and neovascular proliferation [2]. These pathophysiological alterations ultimately disrupt the blood–retinal barrier (BRB) and contribute to inflammatory and neurodegenerative cascades, exacerbating retinal damage. Clinically, DR is categorized into two main stages: nonproliferative DR (NPDR), characterized by early microvascular lesions without new blood vessel formation, and proliferative DR (PDR), which is marked by pathological neovascularization (NV) in response to ischemic retinal damage. With the global prevalence of diabetes steadily rising, the incidence of DR is projected to surge by approximately 55.6% between 2020 and 2045, highlighting the urgent need for advancements in early detection, prognostic biomarker discovery, and therapeutic interventions [3]. Given these alarming trends, an in‐depth investigation into the molecular mechanisms governing DR progression, alongside emerging strategies in epigenetics and biomarker research, is imperative for developing targeted and effective treatments.

Beyond its direct impact on visual function, DR is increasingly recognized as a systemic disease with significant associations with various vascular comorbidities such as cardiovascular disease (CVD), hypertension, chronic kidney disease, as well as cerebrovascular disorders [4–9]. Previous reports describe that the pathophysiology of DR may act as an independent predictor of both all‐cause and cardiovascular‐specific mortality, further elucidating its clinical significance [10–15]. Other reports have demonstrated that patients with DR face a two‐fold higher risk of mortality in Type 2 DM (T2DM) and a four‐fold increase in Type 1 DM (T1DM) compared to those without DR [16]. Furthermore, a more recent analysis revealed that NPDR elevates the risk of all‐cause mortality by 1.38 times, whereas PDR is associated with a 2.32‐fold increase in mortality in individuals with Type 2 diabetes, emphasizing the prognostic implications of DR severity [17]. Existing studies often rely on risk factor adjustments instead of employing direct measures of cardiovascular pathology, such as coronary angiographic evaluations [13, 18], leading to potential confounding in interpretation.

Moreover, conventional approaches to studying DR‐related mortality have primarily dichotomized DR as either present or absent rather than evaluating the impact of varying disease severities on patient outcomes. A more granular analysis of DR severity, incorporating quantitative assessments of vascular and neurodegenerative damage, could provide novel insights into its prognostic value. These limitations highlight the need for more refined research methodologies and advanced imaging modalities to accurately delineate the role of DR in systemic disease progression.

In this review, we provide a comprehensive analysis of the latest advancements in understanding DR pathogenesis, emphasizing the interplay between inflammation, oxidative stress, and neurovascular dysfunction. We also examine the role of multiple signaling pathways, emerging biomarkers in diagnosing and prognosticating DR severity, along with insights from epigenetic studies that shed light on novel regulatory mechanisms underlying DR progression. In addition, we evaluate the efficacy of recent therapeutic innovations, including anti‐VEGF agents, neuroprotective strategies, and regenerative medicine approaches, in both preclinical and clinical settings. By addressing these critical knowledge gaps, this review aims to foster a deeper understanding of DR pathophysiology and facilitate the development of more effective diagnostic and therapeutic approaches for this sight‐threatening and systemic complication of diabetes [19].

### 1.1. Literature Search

This is a narrative review and has provided additional details regarding our literature identification process, including the search terms used, the databases queried, and the timeframe during which the literature search was conducted (e.g., publications from 2000 to 2025). (past 25 years) for literature selection. We also discuss how priority was given to high‐impact articles, particularly those published in leading journals such as Nature, The Lancet, JAMA, Elsevier, and Springer publications, to ensure both depth and relevance of the narrative synthesis. A comprehensive search was performed across several research databases, which include PubMed, Google Scholar, MEDLINE, eMedicine, National Library of Medicine (NLM), and ReleMed, to identify articles pertinent to our research topics. The search process involved both automated searches and manual sorting of selected articles pertinent to this study.

## 2. Pathogenesis of Diabetic Retina and Challenges in Evaluating Diabetic Retina in DR

**FIGURE 1:**
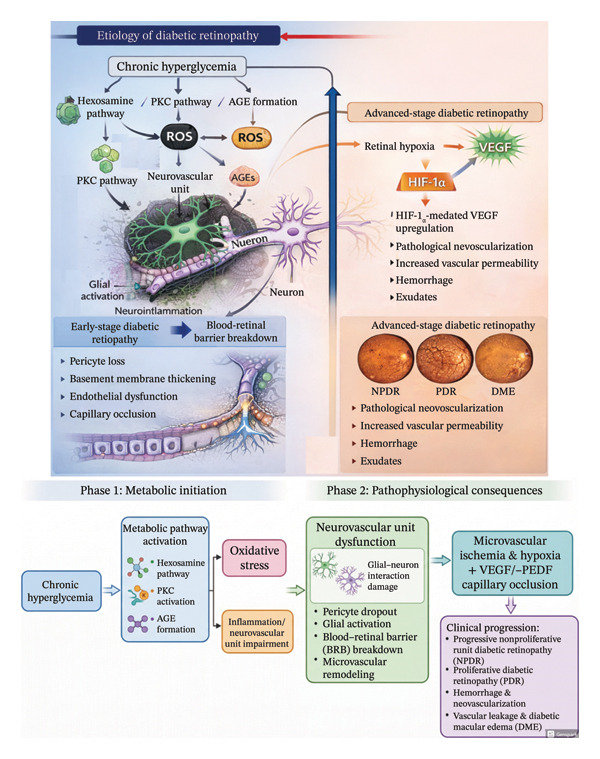
Pathophysiological mechanisms underlying the development and progression of diabetic retinopathy. Chronic hyperglycemia serves as the primary initiating factor in diabetic retinopathy, activating key metabolic pathways such as the hexosamine biosynthetic pathway, Protein kinase C (PKC) signaling, and advanced glycation end‐product (AGE) formation. These interconnected mechanisms promote excessive reactive oxygen species (ROS) generation, mitochondrial dysfunction, and sustained oxidative stress, leading to disruption of the retinal neurovascular unit. Early neuronal and glial alterations, including Müller cell activation and neuroinflammation, contribute to glial–neuronal dysfunction and blood–retinal barrier breakdown. As the disease progresses, cumulative microvascular injury results in pericyte loss, basement membrane thickening, endothelial dysfunction, and capillary occlusion. Retinal ischemia subsequently triggers Hypoxia‐inducible factor‐1α (HIF‐1α)–mediated upregulation of vascular endothelial growth factor (VEGF), promoting pathological neovascularization, increased vascular permeability, hemorrhage, and exudation. These events culminate in advanced‐stage disease, characterized by severe nonproliferative diabetic retinopathy (NPDR), proliferative diabetic retinopathy (PDR), and diabetic macular edema (DME).

Comparative analyses of normal and diabetic retinas, particularly those affected by NPDR with diabetic macular edema (DME), reveal significant structural and functional disparities. In a physiologically healthy retina, vascular integrity is maintained by an intact BRB, comprising the inner and outer layers. This barrier safeguards neuronal elements such as photoreceptors, as well as glial components such as Müller cells and quiescent microglia, ensuring homeostatic retinal function. However, in DR, this delicate microvascular network undergoes progressive pathological alterations, including capillary degeneration, venous beading, microaneurysm formation, and aberrant NV. Concurrently, glial and neuronal dysfunction exacerbates retinal damage, with Müller cell swelling, retinal pigment epithelium impairment, and thinning of the choriocapillaris contributing to disease progression (Figure 1). A compromised BRB facilitates fluid accumulation within the retinal layers, leading to tissue thickening, cystic degeneration, and the presence of subretinal fluid. Collectively, these changes compromise retinal function and visual acuity.

Advancements in ocular imaging over the past years have described critical information into DR pathophysiology, supplementing the traditional seven‐standard field color fundus photography, which has long been the gold standard under Early Treatment DR Study (ETDRS) procedures [20]. Development of ultrawide‐field imaging techniques has significantly expanded the scope of retinal assessment, capturing up to 200° (over 80%) of the retinal surface in a single unique image. This comprehensive visualization enables early detection of peripheral DR lesions, which are correlated with a higher risk of disease progression [21]. Moreover, adaptive optics technology, which compensates for wavefront aberrations, has pushed the boundaries of cellular‐resolution imaging, with the ability to visualize structures as small as 2 μm. This innovation has facilitated detailed studies of the cone photoreceptor mosaic and early vascular abnormalities in diabetic eyes, which remain undetectable using conventional imaging modalities [22, 23].

Optical coherence tomography (OCT) has emerged as a significant procedure for high‐resolution imaging confined to the retina, enabling the differentiation of individual retinal layers and quantitative assessment of retinal thickness. OCT has been instrumental in identifying underlying neuroretinal biomarkers associated with visual acuity in DME, including thinning of the ganglion cell layer, disorganization pertinent to retinal inner layers, and damage to photoreceptors. However, additional validation is required to establish these biomarkers as reliable indicators of disease severity [24–27]. The more recent innovation of OCT angiography (OCTA) has further enhanced retinal imaging by generating detailed perfusion maps of the central retinal vasculature, thereby allowing the identification of early microvascular alterations that contribute to DR progression [28].

In addition to structural imaging, electrophysiological techniques such as full‐field and multifocal electroretinography (ERG) have provided valuable functional insights into DR. These methods reveal disruptions in retinal electrical signaling, with multifocal ERG implicit time changes occurring even before the appearance of microaneurysms or other vascular lesions [29]. Furthermore, visual function assessments, including contrast sensitivity testing, color perception evaluations, frequency‐doubling perimetry, and microperimetry, demonstrate varying degrees of functional impairment corresponding to DR severity. However, these tests lack the specificity and sensitivity needed to serve as standalone predictive biomarkers for DR and DME progression.

Future directions in DR imaging are likely to focus on integrating multimodal imaging approaches to achieve a comprehensive understanding of retinal pathology. Simultaneous or sequential imaging methodologies that selectively target vascular and neural components of the retina may elucidate the chronological sequence of disease progression. Such approaches have the potential to identify novel predictive biomarkers for visual function outcomes, thereby improving risk stratification and facilitating earlier, more effective interventions in DR management [30].

## 3. Lipoproteins and Menopause Implications in DR

T2DM is often accompanied by dyslipidemia, a condition that plays a prominent role in DR pathogenesis by accelerating biochemical and molecular processes, including nonenzymatic glycosylation and activation of the polyol cascade [31, 32]. These processes could further result in the modulation of basement membrane thickness and nonfunction of endothelium and improper function of the retinal barrier, which in turn leads to structural and functional deterioration of the retina [33, 34]. Numerous studies have reported a greater prevalence of DR in diabetic patients with dyslipidemia than those without, suggesting a strong link between lipid imbalances and DR progression [35–37] (Figure 1). Apolipoproteins, predominantly synthesized inside the liver, are essential components of lipid metabolism, facilitating lipid transport and maintaining systemic lipid homeostasis by forming lipoprotein complexes [38, 39]. Recent advancements in DR research suggest that apolipoproteins play a pivotal role in the pathophysiology of DR, influencing both its onset and progression [36]. Key lipid components such as lipoproteins, apolipoproteins, LDL, HDL, and cholesterol have been extensively associated with the incidence of DR [36, 40]. For instance, apolipoproteins could facilitate cholesterol transport to the liver, play a pivotal role in lipid metabolism, and have been implicated as significant contributors to CVDs and DR development [36, 41, 42].

The hormonal changes accompanying menopause significantly impact apolipoprotein levels with the simultaneous prevalence of DR [43, 44]. For example, a sharp decrease in estrogen in postmenopausal women exacerbates dyslipidemia and reduces neuroprotective mechanisms that safeguard retinal ganglion cells, making them more vulnerable to diabetic insults [42, 44, 45].

Despite this well‐documented relationship, the potential interaction between apolipoproteins and menopausal status in DR pathogenesis has not been comprehensively explored. A previous report [42] addressed this gap by investigating the combined effects of apolipoproteins as well as the menopausal status on DR susceptibility in women with T2DM. Previous findings reveal a distinct relationship between apolipoproteins, particularly apoA and apoB, and the prevalence of DR and PDR in women with T2DM, with variations observed between premenopausal and postmenopausal groups [42]. This study elucidates the significance of menopausal status as a critical factor influencing lipid metabolism and its subsequent impact on DR pathogenesis. The incorporation of this variable into predictive models significantly enhanced their accuracy and explanatory capacity [42]. Notably, models integrating apolipoprotein–hormonal interactions exhibited greater predictive power for PDR compared to DR, emphasizing the need to evaluate both lipid profiles and endocrine status when assessing DR risk in women. This integrative approach broadens our understanding of DR mechanisms and offers avenues for personalized risk stratification and targeted therapeutic interventions [42]. This report delineated the differential impact of apolipoproteins on DR risk across premenopausal and postmenopausal populations. The results indicate significant disparities in the association between lipid biomarkers and DR susceptibility. Among premenopausal women, elevated apoA levels (≥ 1.37 g/L) were associated with an increased likelihood of DR, whereas lower apoB levels (≤ 0.78 g/L) correlated with a higher risk of DR. Conversely, in postmenopausal women, an intermediate range of apoA levels was linked to an elevated risk of DR compared to both lower and higher concentrations [42]. Furthermore, postmenopausal individuals with lower apoB levels exhibited a reduced risk of DR relative to those with elevated apoB concentrations. These findings reinforce the clinical importance of routine lipid monitoring in women with T2DM. Specifically, for premenopausal patients, ophthalmologic assessments should be prioritized when apoA levels exceed 1.37 g/L or apoB levels fall below 0.78 g/L [42]. In postmenopausal women, routine fundoscopic examinations are warranted when apoA levels surpass 1.07 g/L or apoB levels exceed 0.98 g/L to facilitate early detection and timely intervention in DR management [42].

As DR is a prevalent microvascular complication of T2DM, affected individuals face an augmented risk of macrovascular comorbidities, including ischemic stroke and coronary artery disease, exacerbating morbidity and mortality rates [9, 46, 47]. Given the severe systemic implications, identifying robust and exact biomarkers for DR remains a paramount clinical priority [42]. This report builds upon and expands prior research, offering novel insights into apolipoprotein‐related DR risk stratification [42]. Another study in India reported an inverse association between HDL and apoAI levels with DR severity, whereas elevated apoB levels exhibited a direct correlation with DR progression in a cohort of DR patients [48]. In addition, a retrospective cohort study involving 1023 diabetic patients demonstrated that high baseline serum apoAI levels conferred a protective effect against DR, whereas elevated apoCIII and apoE levels, along with specific apolipoprotein ratios, correlated with an increased risk of DR [49]. Another report reinforced the negative correlation between serum apoA concentrations and DR risk in T2DM patients [36]. While these studies substantiate the role of apolipoproteins in DR, our research advances the field by integrating the influence of menopause, revealing unique hormonal modulatory effects on lipid metabolism and DR vulnerability.

According to past reports, the apoA and its subtypes exert protective effects against DR, while apoB is positively correlated with the severity of DR. The mechanistic underpinnings of these associations may be attributed to the neuroprotective properties of estrogen, particularly 17β‐estradiol, which plays a crucial role in stabilizing mitochondrial membrane potential in retinal ganglion cells, reducing oxidative stress–induced damage, and modulating apoptotic pathways. Estrogen has been shown to suppress ROS accumulation, upregulate antiapoptotic proteins such as Bcl‐2, and inhibit proapoptotic factors such as Bax, thereby preserving retinal integrity [44]. The protective role of estrogen in premenopausal women may counterbalance the adverse lipid‐mediated effects on DR risk. However, following menopause, the decline in estrogen levels likely exacerbates the detrimental impact of dysregulated apolipoproteins on retinal microvascular health. These findings highlight the necessity of incorporating hormonal considerations into DR risk assessments and emphasize the potential for personalized screening and preventive strategies tailored to menopausal status in women with T2DM [42].

## 4. Blood Omega‐3 Biomarkers and Epigenetics Implications in DR Pathogenesis

DR remains one of the predominant reasons for visual impairment in individuals with T1DM, primarily due to retina’s heightened vulnerability to metabolic and vascular dysfunction. The retina’s high oxygen consumption and reliance on the BRB render it particularly susceptible to microvascular insults. Prolonged hyperglycemia provokes a cascade of inflammatory responses, oxidative stress, and endothelial dysfunction, which collectively contribute to ischemic microvascular disease and retinal neurodegeneration [50]. Given that the structural and functional deterioration of retinal capillaries progresses over time, early identification of these pathological changes could enable timely therapeutic interventions aimed at mitigating DR risk and preventing irreversible vision loss.

Long‐chain omega‐3 (n‐3) polyunsaturated fatty acids (LCn‐3PUFAs), mainly docosahexaenoic acid (DHA) (C22:6n‐3), are crucial components of retinal cell membranes, playing pivotal roles in neuroprotection and vascular homeostasis [51]. Once released from phospholipid bilayers, LCn‐3PUFAs undergo enzymatic conversion into bioactive oxylipins with potent anti‐inflammatory and vascular protection [52, 53]. Given that LCn‐3PUFA levels can be altered through dietary consumption, there has been growing interest in investigating the potential protective effects of omega‐3‐rich diets, particularly fatty fish consumption, in reducing DR incidence.

Higher dietary intake of DHA, alongside eicosapentaenoic acid (EPA) and docosapentaenoic acid (DPA), is linked to good vascular health across the retina in individuals with T1DM [54]. To explore this, the authors assessed circulating omega‐3 levels using gas chromatography, an objective biomarker of omega‐3 intake [55], and examined independent associations with DR prevalence and retinal microvascular health, utilizing OCTA, which is a crucial imaging method to detect retinal perfusion at capillary resolution without the need for contrast dye [56]. OCTA has been validated as a reliable tool for assessing DR severity [54, 57].

Lower omega‐3 biomarker status in T1DM patients suggests that LCn‐3PUFAs play a role in modulating autoimmunity and inflammation, which are implicated in both T1DM development and progression [58]. Preclinical and clinical studies indicate that LCn‐3PUFAs may exert protective effects by modulating immune responses and reducing pancreatic islet autoimmunity [59]. A higher LCn‐3PUFA intake was associated with reduced T1DM risk, as demonstrated through blood biomarker analyses [60–62] and dietary assessments [60, 63]. According to previous studies, independent associations were observed between circulating omega‐3 biomarkers and improved retinal microvascular status. This is particularly relevant given the substantial diabetes‐related vascular complications. While intensive glycemic control remains the cornerstone of DR prevention, growing evidence suggests that dietary factors, including omega‐3 fatty acid intake, may influence DR risk [64]. Furthermore, the application of high‐resolution OCTA imaging enabled us to delineate novel associations between omega‐3 status and retinal vascular health across different stages of DR, a methodology previously applied to diabetic kidney disease [65] and systemic glycemic regulation [57]. To date, limited clinical studies have investigated dietary factors in relation to retinal microvascular integrity using OCTA; one notable study linked higher consumption of vegetables, fruits, fish, and vegetable oils with preserved retinal neurovascular health [66].

A differential impact of omega‐3 subtypes on DR progression was also observed in the previous studies (Table 1). Notably, higher EPA levels could cause a decreased prevalence of DR, particularly in female patients. [70]. Meanwhile, increased levels of longer‐chain omega‐3s (DPA and DHA) were correlated with enhanced macular perfusion. These findings suggest that LCn‐3PUFAs exert distinct but complementary effects on retinal vascular health. EPA may primarily function as a systemic anti‐inflammatory agent, while DHA and DPA may confer direct vascular protection benefits by mitigating central retinal ischemia [70]. This hypothesis warrants further investigation in longitudinal studies [71]. Previous studies provided valuable insights into the interplay between omega‐3 fatty acids and DR risk in T1DM, supporting the potential role of marine‐derived omega‐3s as a modifiable dietary factor in DR prevention [71]. In conclusion, higher circulating levels of omega‐3 fatty acids, particularly EPA, DPA, and DHA, are associated with a lower prevalence of DR and better‐preserved retinal microvascular status in patients with T1D, as measured by OCTA. These results align with experimental models of DR pathogenesis and reinforce the concept of omega‐3 fatty acids as protective agents in T1D‐related vascular complications. Further prospective studies and randomized trials are essential for establishing causality and exploring the therapeutic potential of omega‐3 supplementation in DR prevention [54].

**TABLE 1:** Key biomarkers and epigenetic factors implicated in DR pathogenesis.

Biomarker or miRNA	Epigenetics	Implications of these biomarkers in DR pathogenesis	Implications of epigenetics in DR pathogenesis	Refs
Lipoproteins and apolipoproteins (ApoA, ApoB, LDL, HDL, and cholesterol)	—	Dyslipidemia accelerates biochemical processes such as nonenzymatic glycosylation and polyol cascade, leading to endothelial dysfunction and retinal barrier impairment. Apolipoproteins influence lipid metabolism and DR progression. Menopause alters apolipoprotein levels, affecting DR susceptibility	Menopause‐associated estrogen decline exacerbates dyslipidemia and reduces neuroprotection, worsening DR risk. Estrogen modulates mitochondrial stability and oxidative stress in retinal ganglion cells	[9, 31–39, 41–47]
Omega‐3 fatty acids (DHA, EPA, and DPA)	—	Omega‐3 PUFAs exhibit neuroprotective and vascular benefits. Higher dietary intake correlates with better retinal microvascular health, reducing DR progression. DHA and EPA play distinct protective roles	Omega‐3 PUFAs modulate immune responses, reduce oxidative stress, and protect against retinal ischemia. Blood biomarkers of omega‐3 intake correlate with reduced DR prevalence	[50–71]
miR‐21	Upregulated in DR	Promotes endothelial dysfunction and chronic inflammation, worsening DR	Potential therapeutic target for mitigating endothelial damage in DR	[72]
miR‐216a	Downregulated in DR	Protects retinal microvascular endothelial cells by mitigating NOS2/JAK/STAT signaling, reducing oxidative stress and inflammation	Upregulation may counteract retinal endothelial damage in DR	[73]
miR‐210	Upregulated in DR	Plays a role in vascular endothelial proliferation; serum levels elevated in DR patients, serving as a biomarker for early DR detection	Potential biomarker for distinguishing PDR from NPDR	[74]
miR‐29b‐3p	Upregulated in DR	Induces apoptosis in HRMECs by targeting SIRT1, contributing to microvascular injury in DR	Could serve as a target for preventing endothelial apoptosis	[75]
miR‐203a‐3p	Downregulated in DR	Suppresses VEGF‐A and HIF‐1*α*, inhibiting pathological angiogenesis in DR	Potential therapeutic target for preventing neovascularization in DR	[76]
miR‐320	Downregulated in DR	Chronic hyperglycemia downregulates miR‐320, increasing ET‐1, VEGF, and fibronectin, leading to vascular dysfunction	ERK1/2 pathway involvement in glucose‐induced endothelial damage.	[77]
Blood biomarkers (leukocyte count, urea, bilirubin, AST/ALT, RBC count, and Cystatin C)	—	Increased inflammatory markers and renal dysfunction indicators in DR patients. Lower RBC counts indicate compromised erythropoiesis	Systemic inflammation and metabolic dysregulation contribute to DR pathogenesis	[78]

*Note:* It includes various biomarkers such as lipoproteins, apolipoproteins, omega‐3 fatty acids, and miRNAs, along with their corresponding epigenetic modifications implicated in DR. In addition, the table highlights the impact of epigenetic modifications in DR, emphasizing their influence on gene expression, oxidative stress, and inflammatory pathways.

## 5. MicroRNA (miRNA) as Biomarkers and Epigenetic Implications in DR

miRNAs have emerged as significant biomarkers in various diseases, including DR. Their dysregulation has been implicated in DR pathogenesis, influencing endothelial dysfunction, inflammation, and vascular abnormalities. Among these, miR‐21 is notably upregulated in DR and contributes to endothelial dysfunction induced by DM, leading to chronic low‐grade inflammation, a key factor in DR progression [72]. This suggests that miR‐21 could serve as a potential therapeutic target for mitigating endothelial damage in diabetic patients (Table 1).

Conversely, miR‐216a has been found to exert protective effects to ameliorate damage to human retinal microvascular endothelial cells (HRMECs) in the conditions of DR. It achieves this by mitigating NOS2/JAK/STAT signaling, underlying the inflammatory responses and oxidative stress [73]. This indicates that modulating miR‐216a expression could be a viable strategy for counteracting retinal endothelial damage in DR. Similarly, miR‐210 plays a profound function in vascular endothelial cell proliferation and has been implicated in the development of DR (Table 1), highlighting its significance as a biomarker for early detection of vascular complications in diabetes [74].

In addition, miR‐29b‐3p has been identified as a proapoptotic factor in HRMECs by targeting Sirtuin 1 (SIRT1), a protein crucial for cellular survival and metabolic regulation. This suggests that its upregulation may contribute to the exacerbation of microvascular injury in DR, further emphasizing the role of miRNAs in disease progression [75]. Another important miRNA, miR‐203a‐3p, has demonstrated antiangiogenic properties by suppressing VEGF‐A and HIF‐1α in oxygen‐induced retinopathy models, providing insights into its potential role in inhibiting pathological NV in DR [76].

Serum expression levels of miR‐210 have been reported to be significantly elevated in DR patients compared to both DM patients without retinopathy and healthy controls [74]. This differential expression pattern suggests that serum miR‐210 may serve as a valuable biomarker for distinguishing PDR from NPDR. Furthermore, chronic hyperglycemia has been shown to downregulate miR‐320 while upregulating Endothelin‐1 (ET‐1), VEGF, as well as fibronectin in HUVECs. This dysregulation occurs through the ERK 1/2 pathway, revealing a glucose‐induced mechanism that influences gene expression in endothelial cells [77]. These findings open new objectives to develop new therapeutic modalities to target specific miRNAs to modulate vascular homeostasis and prevent DR progression [79].

## 6. Blood Biomarkers and Physiological Indicators in DR

Despite extensive research on retinal biomarkers in DR, systemic blood‐based physiological indicators remain relatively underexplored. To address this gap, a comprehensive study revealed significant alterations in blood parameters among DR patients compared to controls, suggesting potential systemic biomarkers for DR diagnosis and progression monitoring [78]. Among the key findings, this report described that leukocyte count and urea, including direct bilirubin levels, were markedly elevated in DR patients, reflecting systemic inflammatory responses and potential renal dysfunction associated with advanced diabetes. Interestingly, the ratios of AST to ALT, total bilirubin, indirect bilirubin, and red cell distribution width were significantly decreased in DR patients when compared to the control group, indicating possible hepatic and hematological alterations linked to chronic hyperglycemia [78]. Furthermore, RBC count was typically mitigated in DR patients, suggesting compromised erythropoiesis or increased RBC destruction in DR pathophysiology. In contrast, Cystatin C levels were significantly elevated in DR patients, indicating potential renal impairment, which is a well‐known comorbidity in diabetic microvascular complications [78]. These findings elucidate the importance of systemic physiological markers in DR, which may complement retinal imaging and molecular biomarkers in diagnosing and monitoring disease progression. Given the intricate interplay between systemic inflammation, vascular dysfunction, and metabolic dysregulation in DR, further studies integrating blood biomarkers with miRNA profiling and advanced retinal imaging technologies could enhance the underlying DR pathogenesis and facilitate the development of targeted interventions. The identification of these novel systemic biomarkers could pave the way for personalized medicine approaches, improving early diagnosis, risk stratification, and therapeutic strategies in DR management.

## 7. Implications of Diabetic Retinopathy and CVD Mortality

DR is a major contributor to increased mortality rates among the population. The underlying mechanisms linking DR with increased mortality risk remain incompletely understood, but they appear to extend beyond traditional cardiovascular risk factors. One plausible explanation is that DR serves as an indicator of systemic microvascular dysfunction, which may contribute to the mortality associated with CVD. DR could cause coronary microvascular dysfunction, including diminished coronary flow reserve, impaired blood perfusion to the myocardium, and heightened coronary artery stenosis [80, 81]. These pathological conditions are influenced by chronic inflammation, AGE products, endothelial dysfunction, apoptosis, pathological NV, and oxidative stress, all of which play a critical role in the progression of both microvascular and macrovascular disease [82–85]. A previous report [19] extends this understanding by demonstrating that this association is particularly pronounced in individuals who are at a greater cardiovascular risk. The findings indicate that the presence of any DR typically heightens the risk of CVD‐related mortality among diabetic patients, with a progressively worsening prognosis as the severity of DR advances. Notably, individuals diagnosed with PDR exhibited an almost fivefold‐higher CVD‐related mortality risk than those without DR [19]. Thus, DR serves as an independent predictor of CVD‐related mortality, beyond the influence of baseline cardiovascular risk, preexisting macrovascular complications, and other traditional risk determinants [19]. Interestingly, while the impact of DR on CVD‐related mortality was comparable across genders, the effect was particularly pronounced among individuals aged 65 years and older people than young people [19]. Thus, this study [19] builds upon prior research investigating the interplay between DR and the risk of acquiring CVD‐related mortality. Several epidemiological studies have reported similar findings, reinforcing the independent role of DR in increasing CVD mortality risk [14, 16, 86, 87]. A meta‐analysis encompassing 11,239 diabetic patients reported that individuals with any form of DR had a 1.83‐fold increased risk of CVD mortality, which surged to 2.26 times for those with severe DR [88, 89].

Another potential mechanism is the influence of DR on autonomic nervous system dysfunction, which may exacerbate CVD mortality risk. DR has been implicated in cardiac autonomic neuropathy, a condition associated with a higher risk of mortality because of its adverse effects on cardiovascular regulation. Given these pathophysiological links, DR may not only serve as an early marker of CVD risk but also as an active contributor to adverse cardiovascular outcomes [90–92]. However, previous studies have found that adjusting for these variables has minimal impact on the association between DR and coronary heart disease [9, 13]. In conclusion, DR is a significant, independent predictor of CVD‐related mortality, particularly in individuals with advanced retinopathy. Given its association with microvascular and autonomic dysfunction, DR should be regarded not only as an ocular complication but also as a critical marker of systemic vascular pathology. Future research should explore the potential benefits of early interventions targeting microvascular health in mitigating CVD risk among patients with DR [19].

## 8. Pathophysiology and Pathogenesis of DR Through Multiple Signaling Pathways

**FIGURE 2:**
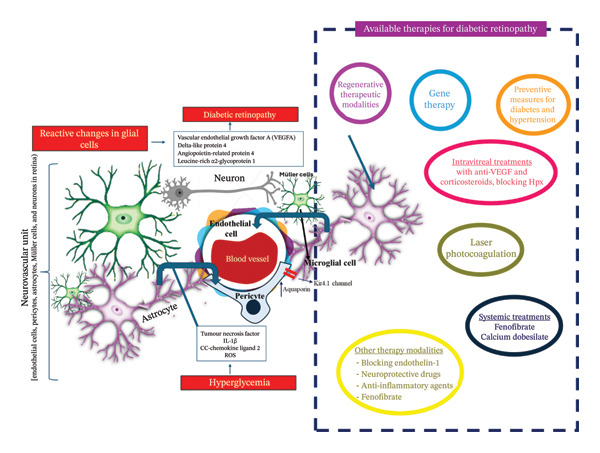
Neuroglial interactions and diabetic retinopathy and advances in therapeutic interventions in diabetic retinopathy (DR): as DR advances, capillary occlusion and retinal ischemia become prominent, marking the transition to preproliferative DR, which is associated with extensive capillary dropout and nonperfused retinal regions. Neurovascular unit (NVU) of the retina, comprising endothelial cells, pericytes, astrocytes, Müller cells, and neurons, plays a crucial role in maintaining retinal homeostasis. In DR, glial cells undergo reactive changes, increasing the expression of aquaporins and Kir4.1 channels, contributing to retinal swelling. In addition, they secrete vasoactive molecules such as Vascular endothelial growth factor A, Delta‐like protein 4, Angiopoietin‐related protein 4, and Leucine‐rich α2‐glycoprotein 1, promoting pathological angiogenesis and vascular permeability. Hyperglycemia‐induced oxidative stress further exacerbates endothelial dysfunction, leading to increased reactive oxygen species (ROS) and proinflammatory cytokines, including TNF‐α, IL‐1β, and CC‐chemokine ligand 2 (CCL2), amplifying retinal damage. Advances in therapeutic strategies for DR: the therapeutic landscape of DR has expanded beyond conventional laser photocoagulation and systemic treatments such as fenofibrate and calcium dobesilate. Intravitreal (IVT) therapies targeting VEGF, including anti‐VEGF agents and corticosteroids, effectively mitigate vascular leakage and neovascularization. Novel regenerative approaches, such as gene therapy and neuroprotective strategies, aim to restore retinal function and NVU integrity. Blocking Endothelin‐1, modulating inflammatory pathways, and targeting oxidative stress are emerging modalities that offer additional therapeutic avenues. Preventive measures, including strict glycemic and blood pressure control, remain fundamental in reducing DR progression and preserving visual function.

DR is a complex disease involving multiple neurovascular signaling pathways beyond the well‐established VEGF pathway. Several alternative signaling mechanisms contribute to vascular permeability and NV, offering novel therapeutic targets (Figures 1 and 2).

### 8.1. Kinin–Kallikrein System in DR Pathogenesis

Recent investigations have highlighted the role of the kinin–kallikrein system in diabetic retinal complications. Elevated levels of Carbonic anhydrase 1 and plasma kallikrein are detected in the vitreous humor of patients with advanced DR [93]. Activation of plasma kallikrein results in kininogen cleavage, generating bradykinin, which binds to its receptors on endothelial cells, triggering vascular permeability. Unlike VEGF, the inhibitors of plasma kallikrein effectively mitigate retinal vascular leakage in experimental models of DM, suggesting an independent and promising therapeutic target (Table 2) [94]. Ongoing clinical trials are evaluating plasma kallikrein inhibitors for DME (NCT03466099).

**TABLE 2:** Key signaling pathways implicated in DR and therapeutic interventions.

Signaling pathway	Implication in DR	Therapeutic molecule and efficacy	Study type	Refs
Kinin–kallikrein system	Elevated plasma kallikrein and Carbonic anhydrase 1 (CA1) contribute to retinal vascular leakage	Plasma kallikrein inhibitors reduce retinal vascular permeability	Clinical (NCT03466099)	[93, 94]
ANGPTL4 and neuropilin	ANGPTL4 modulates endothelial permeability via RhoA activation. Neuropilin mediates permeability and vascular dysfunction	Neuropilin‐targeting therapies under investigation for permeability modulation	Preclinical	[95–99]
LRG1 and TGF‐β signaling	LRG1 promotes pathological angiogenesis via TGF‐β signaling and endoglin interaction	Anti‐LRG1 monoclonal antibody (magacizumab) reduces neovascularization	Preclinical and clinical	[100, 101]
Hyperglycemia‐induced epigenetic regulation	Hyperglycemia triggers oxidative stress via NF‐κB‐mediated RAC1 activation, leading to mitochondrial damage	Epigenetic modulators targeting NF‐κB and oxidative stress pathways	Preclinical	[102, 103]
Neurodegeneration and retinal dysfunction	REDD1 downregulates AKT, inducing apoptosis in retinal neurons	REDD1 inhibitors preserve contrast sensitivity and neuroprotection	Preclinical	[104–108]
Endothelin signaling	Endothelin receptor activation affects vascular and neuronal compartments	Endothelin receptor antagonists with dual vascular and neuroprotective benefits	Preclinical	[109]
NRF2 and oxidative stress regulation	NRF2 modulates antioxidant responses, reducing ischemic retinal damage	NRF2 activators mitigate oxidative stress and retinal ganglion cell loss	Preclinical	[110–117]
VEGF pathway	VEGF drives angiogenesis and vascular permeability in DR	Anti‐VEGF agents (bevacizumab, ranibizumab, and aflibercept) reduce neovascularization	Clinical	[118–120]
Angiopoietin/Tie2 axis	Ang‐2 disrupts Tie2 signaling, causing endothelial dysfunction	Faricimab (VEGF‐A and Ang‐2 inhibitor) stabilizes vascular function	Clinical	[121–123]
Inflammatory cytokines	Elevated IL‐6, TNF‐α, and MCP‐1 contribute to inflammation and vascular damage	Intravitreal corticosteroids (dexamethasone, fluocinolone, and acetonide) reduce inflammation	Clinical	[120, 124]
PDGF signaling	Excessive PDGF signaling leads to pericyte loss and vascular instability	PDGF inhibitors (pegpleranib) investigated for pericyte protection	Preclinical and clinical	[120]
Complement system dysregulation	Genetic variants in CFH and CFHR4 contribute to inflammation in DR	Complement inhibitors targeting CFH under investigation	Preclinical	[125]
Neurovascular interactions	Neurodegeneration may precede vascular damage, defining DR subtypes	Neuroprotective strategies tailored to DR subtypes under exploration	Clinical (EUROCONDOR Study)	[126, 127]
Systemic implications and neurodegenerative links	Retinal abnormalities associated with Alzheimer’s and Parkinson’s disease	Retinal imaging as a biomarker for systemic neurodegenerative diseases	Clinical	[128–130]
Cell‐based therapies	Endothelial progenitor and stem cells proposed for retinal repair	CD34+ progenitor and mesenchymal stem cell–based therapies in development	Preclinical	[131–136]

*Note:* This table summarizes key signaling pathways contributing to DR pathogenesis, highlighting their implications, potential therapeutic molecules, and study types. The information elucidates the complexity of DR and the need for multifaceted therapeutic approaches beyond VEGF inhibition.

### 8.2. Angiopoietin‐like protein 4 (ANGPTL4) and Neuropilin in Retinal Permeability Regulation

ANGPTL4 has emerged as a crucial factor in diabetic retinal vascular dysfunction. Initially identified in the aqueous humor of DME patients, ANGPTL4 has been associated with increased endothelial permeability [95]. ANGPTL4‐mediated permeability operates independently of VEGF receptor 2 (VEGFR2) by binding to neuropilin and activating the small G protein RhoA. Interestingly, conflicting findings suggest that ANGPTL4 may also exert protective effects in other vascular conditions by reducing permeability and modulating inflammation [96] (Figure 2). Given these dual roles, further research is needed to clarify its exact contributions to DR pathogenesis. In addition, Semaphorin 3A (SEMA3A), another neuropilin ligand, has been implicated in retinal vascular leakage. Conditional neuropilin knockout studies suggest that targeting neuropilin may be a broad‐spectrum strategy for reducing vascular permeability from multiple sources [97–99] (Table 2).

### 8.3. Leucine‐Rich α2‐Glycoprotein 1 (LRG1) and TGF‐β Signaling in Pathological Angiogenesis in DR

Gene expression analyses have identified LRG1 as a proangiogenic mediator in DR. LRG1 alters TGF‐β signaling by interacting with the co‐receptor endoglin, thereby promoting pathological NV [100, 101]. Studies in Lrg1‐knockout mice reveal a significant reduction in aberrant retinal angiogenesis. Furthermore, administration of an anti‐LRG1 antibody substantially mitigates pathological vascular proliferation. A humanized anti‐LRG1 monoclonal antibody, magacizumab, has been developed as a potential therapeutic agent for pathological retinal neovascular diseases [100, 101].

## 9. Hyperglycemia‐Induced Oxidative Stress and Redox‐Epigenetic Signaling in Diabetic Retinopathy

Emerging evidence suggests that chronic hyperglycemia modulates endothelial function through epigenetic mechanisms, exacerbating oxidative stress. Increased levels of 5‐hydroxy methyl cytosine and NF‐κB‐induced activation of RAC1 have been observed in diabetic endothelial cells. RAC1, a key component of NADPH oxidase 2, contributes to the overproduction of ROS and causes mitochondrial damage [102, 103]. Given the pivotal role of oxidative stress in DR, targeting hyperglycemia‐induced epigenetic alterations could offer a novel therapeutic approach to mitigate retinal damage.

Chronic hyperglycemia is a central driver of oxidative stress in the diabetic retina, triggering a cascade of redox‐mediated events that contribute to microvascular damage, neuronal dysfunction, and disease progression [137]. Excess glucose influx promotes multiple biochemical pathways, including the polyol pathway, advanced glycation end‐products (AGEs), protein kinase C (PKC) activation, and hexosamine pathway flux, which cumulatively increase the generation of ROS such as superoxide and hydrogen peroxide [137]. These ROS are produced via mitochondrial overproduction, NADPH oxidase activation (particularly NOX2/NOX4 isoforms), and autooxidation of glucose intermediates, leading to impaired cellular antioxidant defenses and oxidative injury to retinal capillary cells and neurons [137].

Critically, oxidative stress and redox imbalance not only cause direct cellular damage but also modulate gene expression through epigenetic mechanisms [138]. Key antioxidants and redox regulatory genes, such as those controlled by the Nrf2 signaling axis, are suppressed in diabetes due to altered chromatin states and promoter methylation patterns [138]. Hyperglycemia induces epigenetic modifications of the Keap1–Nrf2 regulatory complex, impairing Nrf2 nuclear translocation and transcriptional activation of ARE‐driven antioxidant genes (e.g., HO‐1, SOD2, and glutamate–cysteine ligase), thereby weakening endogenous ROS detoxification [138].

Recent research also reveals that hyperglycemia‐induced modifications of redox regulatory genes are mediated via DNA methylation, histone modification, and noncoding RNAs, providing a mechanistic link between metabolic stress and persistent oxidative damage (the “metabolic memory” phenomenon) [102]. For example, Rac1, a key regulator of NOX‐mediated cytosolic ROS production, is transcriptionally activated in response to hyperglycemic‐induced changes in promoter hydroxymethylation (5hmC) and enhanced NF‐κB binding, highlighting a direct epigenetic mechanism controlling redox signaling in retinal endothelial cells [102].

Similarly, evidence shows that histone‐modifying enzymes, such as histone deacetylases (HDACs) and lysine‐specific demethylases (e.g., LSD1), respond to redox changes induced by hyperglycemia, altering chromatin accessibility at promoters of antioxidative and proinflammatory genes [139]. Hyperglycemia increases HDAC activity while reducing histone acetyltransferase activity, thereby suppressing protective genes and enhancing the expression of pathogenic mediators such as MMP‐9 and NF‐κB subunits, which further exacerbate oxidative stress and apoptosis in retinal cells [139].

At a broader level, redox signaling intersects with inflammation; oxidative stress enhances NF‐κB transcriptional activity, fueling proinflammatory cytokine production and leukostasis, which compromise the inner BRB and augment vascular permeability, key pathological features of DR [140]. Collectively, these insights support a model where sustained hyperglycemia (1) drives mitochondrial and NADPH oxidase–derived ROS production; (2) alters redox sensors and transcription factors (e.g., Nrf2 and NF‐κB), leading to disrupted antioxidant responses and heightened inflammation; (3) induces epigenetic reprogramming that perpetuates oxidative stress beyond glycemic normalization (metabolic memory); and (4) amplifies vascular and neural damage via ROS‐dependent signaling pathways that converge on apoptosis, angiogenesis, and barrier dysfunction [140].

## 10. Neurodegeneration and Retinal Dysfunction in DR Progression

Although vascular abnormalities dominate DR pathology, increasing evidence suggests that neuronal damage occurs concurrently, or even precedes, observable vascular changes. Apoptosis of retinal neurons, thinning of nerve fiber and ganglion cell layers, and impaired electrophysiological responses have been documented in diabetic models and patients [104, 105]. A key regulator of retinal neurodegeneration, regulated in development and DNA damage response 1 (REDD1), is implicated in hyperglycemia‐induced apoptosis. REDD1 downregulates AKT signaling, facilitating FOXO1‐mediated cell death. Targeted inhibition of REDD1 has demonstrated neuroprotective effects in diabetic animal models, preserving contrast sensitivity and scotopic ERG responses [106–108]. Furthermore, endothelin signaling affects both vascular and neuronal compartments through distinct receptor subtypes, suggesting that endothelin antagonists could have dual protective roles in DR [109].

### 10.1. Neurovascular Interactions and DR Subtypes

The interplay between vascular and neuronal dysfunction in DR remains an area of active investigation. The European Consortium for the Early Treatment of DR (EUROCONDOR) study analyzed retinal function and structure in diabetic patients without overt microvascular disease [126]. Notably, 61% of patients exhibited neurodegenerative changes in the absence of vascular abnormalities, while 32% of patients with mild vascular defects showed no signs of neurodegeneration. These findings suggest potential DR subtypes with distinct pathophysiological trajectories, necessitating tailored therapeutic strategies. Advances in genetic models allowing cell‐type‐specific gene regulation may help elucidate causal relationships between vascular and neuronal alterations in DR [127].

### 10.2. Systemic Implications of DR and Neurodegenerative Diseases

Growing clinical data indicate that DM, particularly T2DM, predisposes patients to neurodegenerative disorders, including dementia [127] and Alzheimer’s disease [128]. Retinal vessel abnormalities have been linked to cognitive impairment [129], and retinal imaging can be considered as a potential biomarker for neurodegenerative conditions. Proteomic studies of the vitreous humor in DR patients have identified alterations in proteins associated with Alzheimer’s and Parkinson’s disease [130], highlighting potential mechanistic overlaps between retinal and cerebral neurodegeneration. However, further investigations are necessary to validate these associations and determine whether targeting retinal pathology could have systemic neuroprotective effects.

The intricate interplay of multiple signaling pathways in DR describes the complexity of its pathogenesis. Beyond VEGF, alternative neurovascular mediators such as the kinin–kallikrein system, ANGPTL4, LRG1, oxidative stress pathways, and neurodegenerative mechanisms contribute significantly to disease progression. A deeper understanding of these pathways offers novel therapeutic opportunities, emphasizing the need for a multifaceted treatment approach. Future research should focus on personalized interventions targeting distinct DR subtypes, leveraging advancements in molecular genetics and neurovascular biology to develop effective, tailored therapies.

Oxidative stress is implicated in DR pathophysiology, with the transcription factor NRF2 serving as a key regulator of antioxidant defenses. NRF2 is normally sequestered by KEAP1, resulting in its degradation. Under oxidative stress, KEAP1‐mediated repression is relieved, allowing NRF2 activation and transcription of cytoprotective genes. NRF2 deficiency exacerbates ischemia‐induced retinal damage and enhances preretinal NV. Studies using cell‐type‐specific NRF2 knockouts have demonstrated that neuronal‐derived NRF2 is crucial for maintaining vascular integrity, partly through the regulation of Semaphorin 6A (SEMA6A) and Notch signaling [110–112]. Therapeutic potential of NRF2 activation in DR: The protective role of NRF2 in DR is further supported by studies in diabetic mouse models, where NRF2 deletion results in increased vascular permeability and visual impairment. In addition, ischemia‐reperfusion models have revealed that NRF2 activation preserves retinal ganglion cell survival and prevents diabetes‐induced vision loss. Pharmacological NRF2 activators have demonstrated efficacy in restoring visual function, suggesting their potential therapeutic utility in DR management. Given the multifaceted role of NRF2 in vascular and neuronal protection, targeting this pathway may offer a promising strategy for mitigating DR progression [113–117].

## 11. Emerging Therapies for DR: Targeting Key Signaling Pathways

DR is characterized by progressive damage to both neuronal and vascular cells in the retina. Current treatments, primarily centered on VEGF‐A inhibition, have demonstrated efficacy, particularly in advanced stages of the disease. However, these therapies require frequent intravitreal injections and may be ineffective for all patients [141, 142]. Given the increasing global prevalence of DM, novel therapeutic strategies targeting alternative signaling pathways involved in DR pathogenesis are crucial. This section explores emerging targeted therapies aimed at modulating key molecular pathways implicated in DR progression (Table 2).

### 11.1. VEGF Pathway and Anti‐VEGF Therapy

VEGF is a profound angiogenesis regulator and vascular permeability, playing a central role in DR progression. Anti‐VEGF agents including bevacizumab, ranibizumab, and aflibercept effectively reduce pathological NV and macular edema [118–120]. Despite their benefits, nearly 50% of patients exhibit suboptimal responses, suggesting the involvement of additional angiogenic and inflammatory mediators [143, 144]. Aflibercept, a soluble decoy receptor, binds VEGF‐A, VEGF‐B, and PlGF with greater affinity compared to native receptors, offering broader angiogenic blockade [124, 145]. However, pan‐VEGF inhibition poses risks of retinal neurodegeneration, emphasizing the need for neuroprotective strategies alongside anti‐VEGF therapy [146–148].

### 11.2. Angiopoietin/Tie2 Signaling Axis: A Novel Therapeutic Target in DR

Angiopoietin/Tie2 signaling pathway governs vascular stability and maturation, complementing VEGF‐mediated angiogenesis. Angiopoietin‐2 (Ang‐2) antagonizes Tie2 activation, promoting endothelial dysfunction and vascular leakage in DR [121, 122]. Faricimab, a bispecific monoclonal antibody targeting both VEGF‐A and Ang‐2, has shown higher efficacy compared to anti‐VEGF monotherapy, significantly improving retinal outcomes in DME [123]. By restoring Tie2 signaling, therapies targeting the Ang/Tie2 axis offer promising avenues for reducing vascular leakage and inflammation in DR (Table 2).

### 11.3. Inflammatory Cytokines and Corticosteroid‐Based Interventions

Chronic inflammation is a major contributor to DR progression, with elevated levels of proinflammatory cytokines such as IL‐6, TNF‐α, and MCP‐1 driving vascular dysfunction [120] (Figure 2). Intravitreal corticosteroids, including dexamethasone and fluocinolone acetonide implants, exhibit potent anti‐inflammatory effects and neuroprotective properties, thereby improving visual outcomes in refractory DME cases [149, 150]. However, complications such as intraocular pressure elevation and cataract formation necessitate careful patient selection and the development of sustained‐release formulations to minimize adverse effects [149] (Table 2).

### 11.4. PDGF and Combination Therapies

PDGF plays a critical function in pericyte recruitment as well as vessel stabilization. In DR, excessive PDGF signaling contributes to pericyte loss, exacerbating vascular leakage and NV. Dual inhibition of VEGF and PDGF pathways using agents such as rinucumab or pegpleranib has demonstrated potential in preclinical models, but clinical trials have yielded mixed results [120, 124] (Table 2). Further investigation into optimal dosing and combination strategies is warranted to enhance therapeutic efficacy.

### 11.5. Oxidative Stress and Neuroprotection in DR

As we discussed above, the therapeutic agents targeting oxidative stress, such as Nrf2 activators and mitochondrial antioxidants, are under investigation for their potential to mitigate retinal neurodegeneration. In addition, neuroprotective compounds such as brimonidine, which modulate neurotrophic signaling, have demonstrated promise in preserving retinal function in experimental DR models [151–153].

### 11.6. Complement System Dysregulation and Genetic Insights: Other Therapies

Recent studies have identified genetic determinants linked to complement factor H (CFH), CFH‐related protein 4 (CFHR4), and B3GNT8 in DR pathogenesis, highlighting their potential as therapeutic targets [125]. Complement system dysregulation contributes to chronic inflammation and microvascular damage, suggesting that complement inhibitors may provide novel treatment avenues for DR.

### 11.7. Emerging Cell‐Based Therapies for Retinal Repair

Endothelial progenitor cells and hematopoietic stem cells have been explored as potential regenerative therapies for DR. While spontaneous retinal reperfusion in DR is rare [131, 132], cell‐based approaches using CD34+ progenitor cells or mesenchymal stem cells aim to enhance vascular repair and restore retinal perfusion [133–136]. Further studies are needed to optimize cell delivery methods and ensure long‐term efficacy.

Despite significant advances in DR management, current treatments remain limited in their ability to fully address the multifactorial nature of the disease. Targeting alternative signaling pathways, including Ang/Tie2, inflammatory cytokines, oxidative stress, and complement system dysregulation, offers promising therapeutic potential. Future research should focus on personalized treatment strategies, combining anti‐VEGF agents with emerging targeted therapies to improve outcomes for patients with DR [117].

### 11.8. Other Advances in Treatment Approaches

#### 11.8.1. Comparative Efficacy of Panretinal Photocoagulation (PRP) and Intravitreal Conbercept for Proliferative Diabetic Retinopathy

Recent advancements in the management of PDR have explored the synergistic effects of combining PRP with intravitreal anti‐VEGF therapy (Figure 1). A prospective clinical investigation assessed the efficacy of PRP alone versus PRP combined with intravitreal conbercept injections in 15 patients diagnosed with PDR [154]. Each participant was randomly assigned to receive PRP in one eye, while the other eye received a combination of PRP and conbercept. The results demonstrated that the combination of PRP and conbercept significantly reduced NV leakage area compared to PRP alone, with notable improvements observed at three and six months posttreatment. In addition, the combination therapy led to superior best‐corrected visual acuity (BCVA) during the initial 3 months. Intragroup analyses corroborated a significant decline in NV leakage at three and six months in both cohorts, with a marked improvement in BCVA observed within 1 month in the combinatorial regimen group. These findings elucidate the therapeutic advantage of integrating intravitreal conbercept with PRP in effectively mitigating NV and enhancing visual outcomes in PDR patients [154].

#### 11.8.2. Genetically Determined Plasma Proteins as Novel Drug Targets in DR

Recent investigations have identified a potential genetic basis for DR through the analysis of plasma protein levels. Specifically, studies have established a causal link between genetically determined concentrations of CFH, CFHR4, and beta‐1,3‐N‐acetylglucosaminyltransferase 8 (B3GNT8) with DR. These proteins may play a significant role in DR pathogenesis by modulating inflammatory and complement pathways, as well as glycosylation processes crucial for vascular integrity. The identification of these molecular targets provides promising avenues for the development of novel pharmacological interventions aimed at modifying disease progression. However, further reports are necessary to elucidate the precise mechanisms by which these proteins lead to DR pathophysiology and to validate their potential as therapeutic targets in clinical settings [125] (Table 2).

#### 11.8.3. Postoperative Intravitreal Bevacizumab (IVB) for Neovascular Glaucoma (NVG) Prevention in PDR Patients Undergoing Phacovitrectomy

NVG remains a severe complication following phacovitrectomy in patients with PDR, necessitating the exploration of prophylactic strategies. A recent clinical study evaluated the efficacy of postoperative IVB in reducing the incidence of NVG and identified risk factors contributing to its development [155]. Findings of this report highlight the protective role of postoperative IVB in reducing NVG incidence and suggest that early intervention with IVB may be beneficial in high‐risk PDR patients undergoing phacovitrectomy [155].

The evolving landscape of DR management describes the necessity for innovative therapeutic approaches beyond conventional treatments. The integration of PRP with intravitreal conbercept offers superior vascular stabilization and visual acuity outcomes compared to PRP alone. The identification of genetically determined plasma proteins as potential therapeutic targets highlights new opportunities for molecular interventions aimed at halting DR progression. Furthermore, the prophylactic administration of IVB postphacovitrectomy demonstrates significant promise in preventing NVG, particularly in high‐risk populations. Future research should focus on validating these strategies in larger clinical trials and refining personalized treatment modalities to optimize patient outcomes in DR management.

## 12. Conclusion and Future Directions

DR remains a leading cause related to vision impairment and blindness among individuals with diabetes, necessitating continuous advancements in understanding its pathogenesis, biomarkers, and therapeutic strategies. This review has provided a comprehensive evaluation of the molecular mechanisms underlying DR, particularly emphasizing the role of lipoproteins, omega‐3 biomarkers, and epigenetic modifications such as miRNAs in disease progression. In addition, the intricate interplay between hyperglycemia‐induced oxidative stress, neurovascular dysfunction, and systemic complications highlights DR as not merely an ophthalmic condition but a systemic vascular disorder with profound implications for overall health, including CVD and neurodegenerative disorders.

A significant challenge in DR management lies in the accurate evaluation of diabetic retina and the identification of reliable biomarkers that can facilitate early detection, prognostication, and personalized therapeutic interventions. Advances in multiomics technologies, including proteomics, metabolomics, and epigenomics, hold promise for identifying novel molecular signatures predictive of DR progression and treatment response. Furthermore, the integration of artificial intelligence (AI) and deep learning in retinal imaging offers unprecedented opportunities for automated, real‐time diagnosis, thereby improving screening efficiency and accessibility.

Emerging therapies targeting key signaling pathways implicated in DR pathogenesis, such as VEGF, inflammatory cytokines, and oxidative stress regulators, have shown considerable potential in both preclinical and clinical settings. While anti‐VEGF agents such as conbercept and bevacizumab have demonstrated efficacy in reducing NV and preserving visual function, their long‐term efficacy and safety warrant further investigation. Moreover, novel therapeutic modalities, including gene therapy, RNA‐based interventions, and nanoparticle‐based drug delivery systems, represent promising frontiers for precision medicine in DR treatment.

Future research should focus on elucidating the role of genetic predisposition and environmental factors in DR susceptibility, with an emphasis on personalized medicine approaches. The interplay between gut microbiota dysbiosis, systemic inflammation, and retinal neurodegeneration is another emerging area warranting exploration, as it may provide novel therapeutic targets. In addition, longitudinal cohort studies assessing the long‐term efficacy of combination therapies, including anti‐VEGF agents, corticosteroids, and neuroprotective drugs, are essential to refine treatment paradigms and improve patient outcomes.

In conclusion, while substantial progress has been made in understanding DR pathophysiology and therapeutic strategies, further interdisciplinary research is essential to bridge the gap between benchside discoveries and clinical applications. The integration of molecular diagnostics, AI‐driven retinal screening, and innovative therapeutics will be pivotal in transforming DR management, ultimately reducing the global burden of diabetic vision loss.

## Author Contributions

Zixuan Huang made a significant contribution to the conception, study design, execution, acquisition of data, analysis, and interpretation, or in all these areas. Zixuan Huang, Jing Wei, and Dayong Yang have drafted or written, substantially revised, or critically reviewed the article. Dayong Yang has agreed on the journal to which the article will be submitted.

## Funding

The authors have nothing to report.

## Disclosure

All the authors reviewed and agreed on all versions of the article before submission and during revision, and the final version was accepted for publication, and any significant changes were introduced at the proofing stage. All the authors agreed to take responsibility and be accountable for the contents of the article. All the authors read and approved the submission of this manuscript.

## Ethics Statement

The authors have nothing to report.

## Consent

The authors have nothing to report.

## Conflicts of Interest

The authors declare no conflicts of interest.

## Data Availability

Data sharing is not applicable to this article, as no new data were generated or analyzed in this study.
